# PAR1 participates in the ability of multidrug resistance and tumorigenesis by controlling Hippo-YAP pathway

**DOI:** 10.18632/oncotarget.5858

**Published:** 2015-09-28

**Authors:** Daisuke Fujimoto, Yuki Ueda, Yasuo Hirono, Takanori Goi, Akio Yamaguchi

**Affiliations:** ^1^ First Department of Surgery, Faculty of Medicine, University of Fukui, Fukui, Japan

**Keywords:** gastric cancer, PAR1, Hippo-YAP pathway, tumorigenesis, drug resistance

## Abstract

The Hippo pathway significantly correlates with organ size control and tumorigenesis. The activity of YAP/TAZ, a transducer of the Hippo pathway, is required to sustain self-renewal and tumor-initiation capacities in cancer stem cells (CSCs). But, upstream signals that control the mammalian Hippo pathway have not been well understood. Here, we reveal a connection between the Protease-activated receptor 1 (PAR1) signaling pathway and the Hippo-YAP pathway in gastric cancer stem-like cells. The selective PAR1 agonist TFLLR-NH_2_ induces an increase in the fraction of side population cells which is enriched in CSCs, and promotes tumorigenesis, multi cancer drug resistance, cell morphological change, and cell invasion which are characteristics of CSCs. In addition, PAR1 activation inhibits the Hippo-YAP pathway kinase Lats via Rho GTPase. Lats kinase inhibition in turn results in increased nuclear localization of dephosphorylated YAP. Furthermore, PAR1 activation confers CSCs related traits via the Hippo-YAP pathway, and the Hippo-YAP pathway correlates with epithelial mesenchymal transition which is induced by PAR1 activation. Our research suggests that the PAR1 signaling deeply participates in the ability of multi drug resistance and tumorigenesis through interactions with the Hippo-YAP pathway signaling in gastric cancer stem-like cells. We presume that inhibited YAP is a new therapeutic target in the treatment human gastric cancer invasion and metastasis by dysregulated PAR1 or its agonists. The Hippo pathway significantly correlates with organ size control and tumorigenesis. The activity of YAP/TAZ, a transducer of the Hippo pathway, is required to sustain self-renewal and tumor-initiation capacities in cancer stem cells (CSCs). But, upstream signals that control the mammalian Hippo pathway have not been well understood. Here, we reveal a connection between the Protease-activated receptor 1 (PAR1) signaling pathway and the Hippo-YAP pathway in gastric cancer stem-like cells. The selective PAR1 agonist TFLLR-NH_2_ induces an increase in the fraction of side population cells which is enriched in CSCs, and promotes tumorigenesis, multi cancer drug resistance, cell morphological change, and cell invasion which are characteristics of CSCs. In addition, PAR1 activation inhibits the Hippo-YAP pathway kinase Lats via Rho GTPase. Lats kinase inhibition in turn results in increased nuclear localization of Dephosphorylated YAP. Furthermore, PAR1 activation confers CSCs related traits via the Hippo-YAP pathway, and the Hippo-YAP pathway correlates with epithelial mesenchymal transition which is induced by PAR1 activation. Our research suggests that the PAR1 signaling deeply participates in the ability of multi drug resistance and tumorigenesis through interactions with the Hippo-YAP pathway signaling in gastric cancer stem-like cells. We presume that inhibited YAP is a new therapeutic target in the treatment human gastric cancer invasion and metastasis by dysregulated PAR1 or its agonists.

## INTRODUCTION

Neoplasm is an abnormal growth in tissues and organs which undergo constant remodeling and regeneration. As these processes are normally maintained by stem cells, this has led to the attractive hypothesis that tumor initiation and progression are also driven by cancer stem-like cells (CSCs). CSCs are defined as those cells that can generate tumors through the stem cell processes of self-renewal and differentiation into multiple cell types [1].

Side population is a flow cytometry (FCM) term to define cell clusters with strong ability to efflux DNA dye Hoechst 33342 via ABC-transporters [2]. Side population cells have been widely reported to be enriched in various cancerous tissues such as breast cancer [3], gastrointestinal system tumor [4], and small-cell lung cancer [5] as well as from cell lines such as nasopharyngeal carcinoma [6], hepatocellular carcinoma [7], and bladder cancer cell lines [8]. Remarkably, side population cells, with stemness potential, can form xenograft tumors in animals and are resistant to chemotherapy and radiotherapy, contributing to tumor relapse [9]. The cancer side population cells are highly enriched with tumorigenic stem-like cancer cells [10]. And side population cells of pancreatic cancer predominated in TGF-beta induced epithelial to mesenchymal transition (EMT) and invasion, but Main population cells did not respond to TGF-beta induced invasion [11]. These findings suggest that side population cells are strongly correlated to EMT in cancer cell.

The Hippo pathway was initially defined by genetic studies in Drosophila, wherein mosaic mutations of Hippo pathway genes resulted in tissue overgrowth [12]. And its core components in organ size regulation were evolutionally conserved in mammals [13]. In the Hippo pathway, a conserved kinase cascade in which the protein kinase MST1/2 phosphorylates and activates the Lats1/2 kinase functions to inhibit YAP and TAZ transcription co-activators by phosphorylation. Dephosphrylated YAP/TAZ localize in the nucleus and function as transcription co-activators for the TEAD family to induce gene expression, including connective tissue growth factor and Cyr61, thereby promoting cell growth, proliferation, and survival [14-16]. Several recent studies have clearly established a role of the Hippo pathway in regulating cell contact inhibition, organ size control, and cancer development [17-19]. In addition it is essential both during developmental growth and in maintaining homeostasis of adult organs, and when deregulated it can cause tumorigenesis [20]. The Hippo pathway has major roles in the modulation of cell proliferation, apoptosis, migration, and differentiation.

We showed activated Protease-activated receptor-1 (PAR1), which is a G-protein coupled receptor (GPCR) and activated when thrombin cleaves the amino-terminal extracellular domain at a specific site, promoted cell invasion and induced Snail mediated E-cadherin transcriptional repressor and ultimately EMT in gastric cancer cells [21, 22]. In this study, we investigated the transformation into CSCs by activated PAR1 and the functional relationship between activated PAR1 signaling and Hippo-YAP regulation in gastric cancer. We show that stimulation of PAR1 by agonist peptide TFLLR-NH_2_ activates YAP by dephosphorylation and activation of YAP increases its nuclear localization which in turn increases the rate of side population cell formation, which is enriched with CSCs. Gastric cancer cell lines acquire multidrug resistance and tumorigenesis by PAR1 activation. As well as, PAR1 acts through RhoA, Lats and YAP to stimulate gene expression of cell migration. Our study indicates a novel function of PAR1 in Hippo pathway regulation and activated PAR1 confers CSCs traits in gastric cancer.

## RESULTS

### Activated PAR1 has potential to induce gastric cancer cell to side population subpopulation

**Figure 1:**
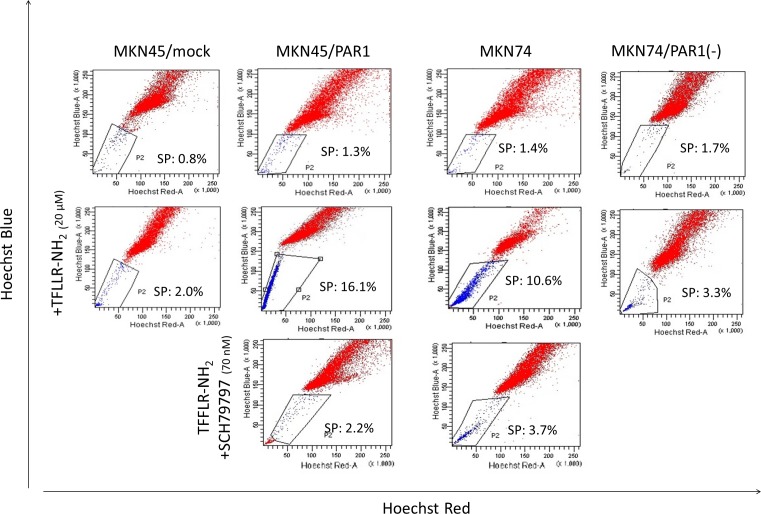
Representative FACS analysis of side population cells in human gastric cancer cell lines The upper row; these panels are FACS analysis of side population MKN45/mock, MKN45/PAR1, MKN74, and PAR1 siRNA transfected MKN74 (MKN74/PAR1(−)) cells which were not stimulated. The middle row; these panels are FACS analysis of side population MKN45/mock, MKN45/PAR1, MKN74, and MKN74/PAR1(−) cells which were treated with the PAR1 agonist TFLLR-NH_2._ The side population fractions in MKN45/PAR1 and MKN74 cells treated with TFLLR-NH_2_ were increased 16.1% and 10.6%, respectively. The lower row; these panels are FACS analysis of side population MKN45/PAR1 and MKN74 cells which were treated with TFLLR-NH_2_ and PAR1 selective antagonist SCH79797.

The side population phenotype as identified by low Hoechst 33342 blue/red fluorescence intensity was detected in 1-3 % of MKN45/mock cells. The side population was detected in 10-16% of MKN45/PAR1 and MKN74 cells which were treated with TFLLR-NH_2_ 24 h. But the loss of the side population was for MKN45/PAR1 and MKN74 cells with TFLLR-NH_2_ either by addition of SCH79797 or knockdown of PAR1 gene by PAR1 siRNA (Figure 1). PAR1 knockdown MKN74 cell by transfected PAR1 siRNA was shortened to MKN74/PAR1(−) (Figure S1).

### Activated PAR1 induced tumor-initiating potential and spheroid colony formation

**Figure 2:**
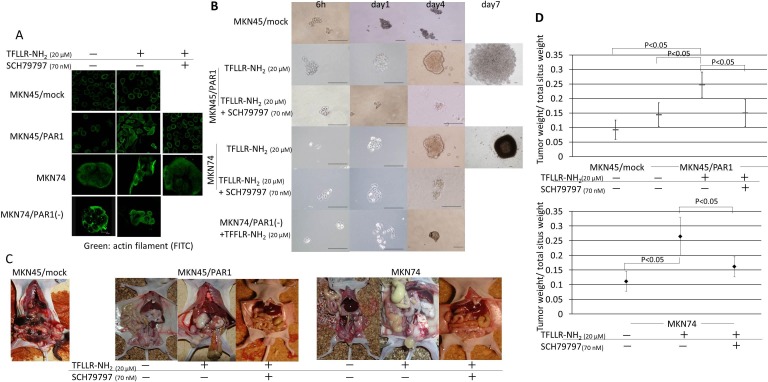
PAR1 activation induces tumor initiating potential in gastric cancer cells **A.** Immunofluorescence staining with anti-actin (green) in gastric cancer cells. MKN45/PAR1 and MKN74 cells which were treated with TFLLR-NH_2_ presented morphological change. **B.** Spheroid colony formation assay. MKN45/mock, MKN45/PAR1, MKN74, and MKN74/PAR1(−) cells were cultured in ultra-low attachment microplates under the various conditions, and both MKN45/PAR1 and MKN74 cells treated with TFLLR-NH_2_ were cultured for 7 days and other conditions cells were cultured for 4 days. All scale bars is 100 μm. **C.** Evaluation of tumor proliferation in nude mice revealed extensive peritoneal dissemination in MKN45/PAR1 and MKN74 cells treated with TFLLR-NH_2_. **D.** Effect of PAR1 activation on primary tumor growth and spread. Before inoculation of MKN45/PAR1 and MKN74 cells, these cells treated with TFLLR-NH_2_ and/or SCH79797. The groups were compared to each other with respect to tumor burden (given as the ratio of tumor weight over total situs weight). The median value is indicated by a bold bar.

Both MKN45/PAR1 and MKN74 cells, which were treated with TFLLR-NH_2_, presented morphological change in the monolayer conditions (Figure 2A), and formed many large spheroid colonies when cultured in ultra-low attachment conditions (Figure 2B). However, MKN45/mock cells did not change their cellular morphology. Cellular morphology was also unchanged for cultures of MKN45/PAR1 and MKN74 cells in which PAR1 activity was suppressed by the addition of SCH79797 or siRNA (Figure 2A). In addition, these cells formed a few small spheroid colonies when cultures in ultra-low attachment conditions (Figure 2B).

With regard to evaluation of tumor proliferation in the nude mice, typically large tumor nodules on the peritoneum, as well as an abundant tumor spread across the peritoneal muscle layer, mesentery and the diaphragm was observed in MKN45/PAR1 and MKN74 cells which were pretreated with TFLLR-NH_2_ group (Figure 2C).

The statistical analysis of the ratios of tumor weight over total situs weight proved that PAR1 activated gastric cancer cell by TFLLR-NH_2_ resulted in a significant increase of tumor burden (MKN45/PAR1 and MKN74 cells treated by TFLLR-NH_2_ versus control, *P* < 0.05; Figure 2D). The peritoneal dissemination tumor weight of MKN45/PAR1 and MKN74 cells treated with TFLLR-NH_2_ plus SCH79797 were small as compared to MKN45/PAR1 and MKN74 pretreated with TFLLR-NH_2_ alone (*P < 0.05*; Figure 2D).

### Activated PAR1 induced anti-cancer drug resistance

**Figure 3:**
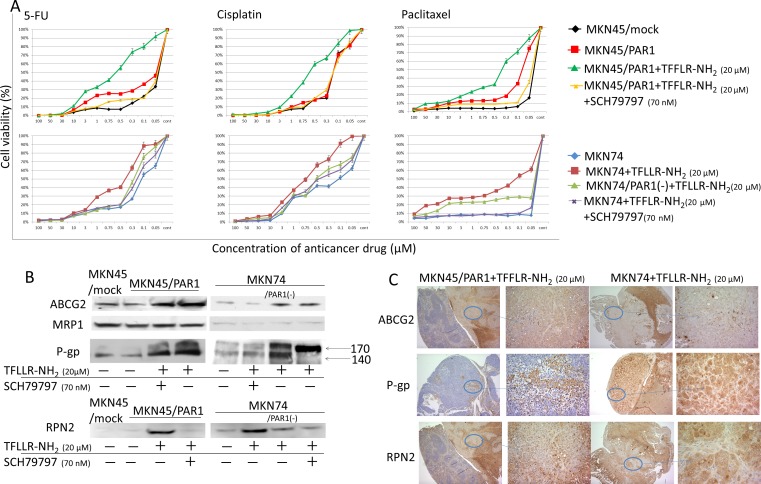
PAR1 activation acquires anti-cancer drug resistance **A.** Cell viabilities in response to chemotherapeutic induction, and the degrees of cytotoxicity of Cisplatin, 5-FU and Paclitaxel on the cells were measured. Under various conditions, MKN45/mock, MKN45/PAR1 and MKN74 cells were seeded in 96-well plates and treated with Cisplatin, 5-FU, and Paclitaxel for 24 h prior to the determination of cell viability. Data are the mean ± SD of five independent experiments. **B.** Western blot assays showing ABC-transporter, ABCG2, MRP1 and P-gp expression, and RPN2, which regulated glycosylation status of P-gp, by western blot assays. **C.** We show peritoneal dissemination in nude mice indicated by means of immunhistochemical staining of ABCG2, P-gp, and RPN2.

Cell viabilities in response to chemotherapeutic induction, and the degrees of cytotoxicity of Cisplatin, 5-FU and Paclitaxel on the cells were measured using cell counting kit CCK-8. Cisplatin, 5-FU and Paclitaxel could evenly decrease the cell viabilities of MKN45/mock, MKN45/PAR1 and MKN74 cells, which were exposed to various conditions, in a dose-dependent manner. Both MKN45/PAR1 and MKN74 cells treated with TFLLR-NH_2_ showed drug resistance compared to control of these cells (*P < 0.05*; Figure 3A). But both MKN45/PAR1 and MKN74 cells with PAR1 activity suppressed by SCH79797 or PAR1 siRNA presented an inhibition of resistance to these drugs (Figure 3A).

Next, we observed ATP-binding cassette transporter (ABC-transporter), ABCG2, MRP1 and P-gp expression by means of western blot assay. MKN45/PAR1 and MKN74 cells which were treated with TFLLR-NH_2_ presented increased expression levels of ABCG2 relative to cultures of these cells in which PAR1 activation was suppressed or PAR1 was the non-stimulating condition (Figure 3B). We profiled RPN2 expression by western blotting assay. RPN2 increases glycosylation status of P-gp. And when P-gp is glycosylated, it has been shown to take part in drug egest [23]. We profiled the glycosylation patterns by means of western blots in which glycosylated P-gp, appears on blots as mature 170-kDa bands and un-glycosylated P-gp as 140-kDa bands. The 140-kDa unglycosylated P-gp was clearly found in MKN45/mock cells (Figure 3B). In MKN45/PAR1 and MKN74 cells which PAR1 activation was supressed by SCH79797 or PAR1 siRNA transfected, unglycosilated P-gp was found, and trace amounts of glycosylated P-gp was found just a little (Figure 3B). In contrast, MKN45/PAR1 and MKN74 cells treated by TFLLR-NH_2_ expressed glycosylated 170-kDa matured P-gp (Figure 3B). Similarly, immunohistochemistry indicated that ABCG2, P-gp, and RPN2 were highly expressed in the peritoneal dissemination mass in nude mice (Figure 3C).

### PAR1 stimulation induces YAP dephosphorylation and nuclear localization

**Figure 4:**
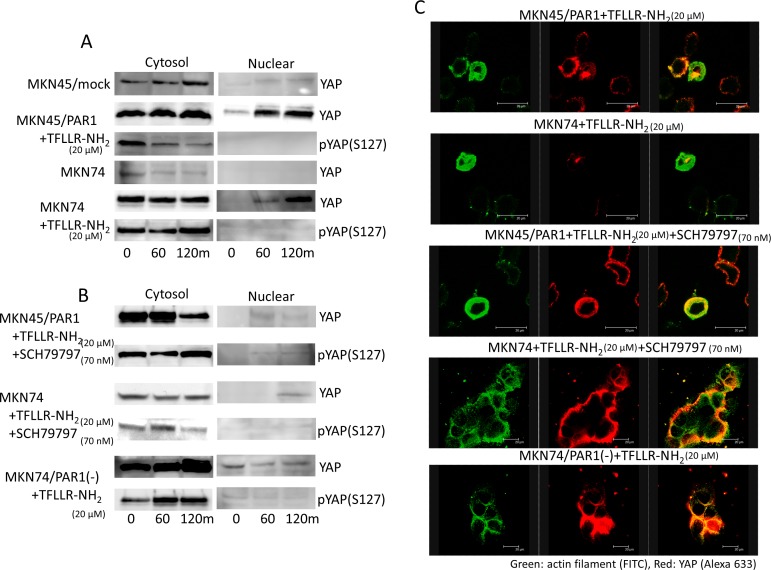
PAR1 promotes YAP dephosphorylation The left line panels are immunoblotting of cytoplasmic lysates. The right panels are immunoblotting of nucleus lysates. **A.** Both MKN45/PAR1 and MKN74 cells were treated with TFLLR-NH_2_ for indicated periods of time. Cytoplasmic and nuclear cell lysates were separated. And these cells lysates were subjected to immunoblotting with the YAP1 and pYAP1 (S127). **B.** Both MKN45/PAR1 and MKN74 cells were treated with TFLLR-NH_2_ and SCH79797 for indicated times. MKN74/PAR1(−) cells were treated with TFLLR-NH_2_ for indicated times. These cell lysates were also separated cytoplasmic and nucleus and subjected to immunoblotting with the YAP1 and pYAP1 (S127). **C.** Immunofluorescence staining with anti-actin (green) and anti-YAP1 (red) antibodies; the right panels show the overlay of the green and red staining.

In search of Hippo-YAP signalling pathway, we found that YAP was much more abundant in the cytoplasmic lysate relative to the slight amounts observed in the nuclear lysate in MKN45/mock and MKN74 cells (Figure 4A). But addition of TFLLR-NH_2_ to MKN45/PAR1 and MKN74 cells resulted in a rapid decrease in cytoplasmic and phosphorylated YAP, and increase in nuclear and dephosphorylated YAP as indicated by western blot assays (Figure 4A). Immunofluorescence assay indicated that Dephosphorylated YPA moved into the nucleus under conditions in which PAR1 activity was stimulated (Figure 4C). Under conditions of supressed PAR1 activation of MKN45/PAR1 cells, we found that phosphorylated YAP was highly abundant in the cytoplasmic lysate and YAP did not move into the nucleus (Figure 4B and 4C). Similarly, under conditions in whichPAR1 activation was supressed in MKN74 cells, YAP did not move into the nucleus as indicated by western blot and immunofluorescence assays (Figure 4B and 4C).

### TFLLR-NH2 increases YAP dephosphorylation not *via* a Rho kinase but *via* a Rho

**Figure 5:**
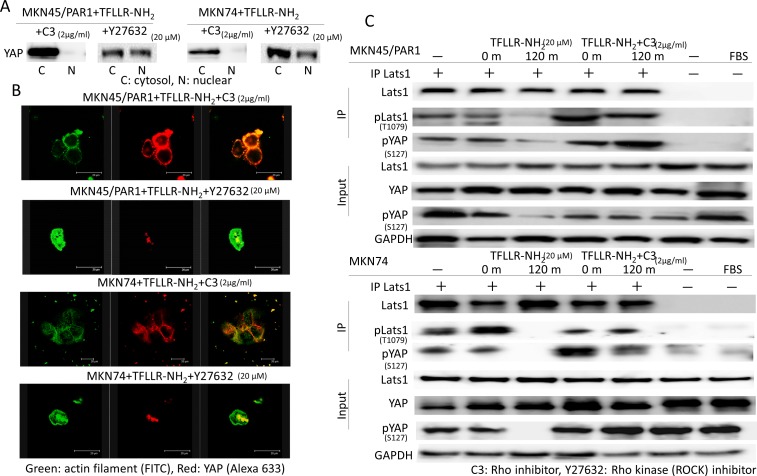
A Rho inhibitor, C3 repress YAP activity **A.** Inactivation of Rho by C3 prevents YAP1 dephosphorylation caused by TFLLR-NH_2_. Whereas, a ROCK inhibitor, Y27632 never prevented YAP1 activation induced by TFLLR-NH_2_. **B.** Differential immunofluorescence imaging of actin filament and YAP1 proteins. C3 inhibited YAP1 nuclear localization. But Y27632 was not able to control YAP1 nuclear localization. **C.** Lats kinase activity is inhibited by C3. Both MKN45/PAR1 and MKN74 cells were previously transfected with the pGEX-KG-GST-YAP. MKN45/PAR1 and MKN74 cells pretreated with C3 for 5 h and then incubated with TFLLR-NH_2_ for 2 h. The presence of FBS is indicated. Last1 immunoprecipitated from the cell lysates was subjected to *in vitro* kinase assays using GST-YAP as a substrate. YAP1 phosphorylation was detected by pYAP1 (S127) antibody. Phosprylated Lats1 was detected by pLats1 (T1079) antibody.

We reported that activated PAR1 induced Rho GTPase activation, and Rho GTPase has been reported to induce YAP dephophorylation [24, 25]. We analysed the function of Rho in TFLLR-NH_2_-induced YAP dephosphorylation. To determine the function of Rho in YAP regulation, we tested the effect of botulinum toxin C3 (for 5 h), a specific inhibitor of Rho GTPase, and Y27632 (for 4 h), Rho-associated kinase (ROCK) inhibitor, on YAP phosphorylation. Western blot assays indicate that C3 treatment strongly suppressed YAP dephosphorylation in both MKN45/PAR1 and MKN74 cells treated with TFLLR-NH_2_ (Figure 5A). Immunofluorescence assays of both MKN45/PAR1 and MKN74 cells treated with TFLLR-NH_2_ and C3 indicate that YAP did not moved into the nucleus (Figure 5B). In contrast, Inhibition of ROCK by Y27632 treatment had a marginal effect on TFLLR-NH_2_ induced YAP dephosphorylation and nuclear localization (Figure 5A and 5B).

Lats1/2 forms a cascade to increase YAP phosphorylation [26]. We analyzed Lats1 kinase activity to determine whether Lats1/2 kinase is involved in YAP regulation. TFLLR-NH_2_ treatment resulted inhibition of Lats1 kinase and C3 treatment blocked TFLLR-NH_2_ induced inhibition of Lats1 kinase (Figure 5C). Additionally, there is an observed relationship between Lats1 inactivation and YAP phosphorylation (Figure 5C).

### Hippo-YAP pathway promotes cancer cell migration and morphology change, and maintained cancer stem-like cell

**Figure 6:**
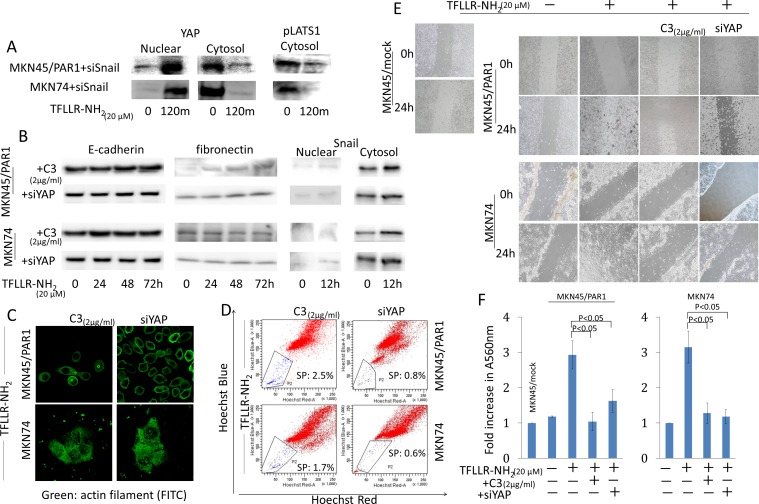
Hippo-YAP pathway caused by PAR1 activation promotes EMT, and ensures the preservation of cancer stem-like cell **A.** Even when Snail was inhibited by siRNA, PAR1 activation promoted dephophorylation of YAP1 and inhibited phosphorylation of Lats1. YAP1 and pLats1 (T1079) were detected by western blot assays. **B.** The cytosolic and nuclear expression levels of E-cadherin, fibronectin and Snail were detected by western blot assays. MKN45/PAR1 and MKN74 cells were pretreated with C3 for 5 h or YAP1 was knocked down by siRNA and then incubated with TFLLR-NH_2_ for indicated times. **C.** Immunofluorescence staining of anti-actin (green) shows the morphology of MKN45/PAR1 and MKN74 cells, which were pretreated with C3 for 5 h or YAP1 was knocked down by siRNA, were unchanged. **D.** FACS analysis of side population showed C3 treatment and knockdown of YAP1 efficiency blocked increase side population cells, even when MKN45/PAR1 and MKN74 cells were treated with TFLLR-NH_2_. **E.** MKN45/PAR1 and MKN74 cell cultures, which were pretreated with C3 for 5 h or YAP1 was knocked down by siRNA, filled in a scratch made with 200-μl pipette tip after 24 h similar to control cells, and MKN45/PAR1 and MKN74 cells treated with TFLLR-NH_2_ recovered more efficient than control cells. **F.** Invasion assay shows MKN45/PAR1 and MKN74 cells, which were pretreated with C3 for 5 h or YAP1 was knocked down by siRNA and then incubated with TFLLR-NH_2_, presented a significant decline in invasion potential relative to these cells treated with TFLLR-NH_2_ only. A560 nm of MKN45/mock and MKN74 cultured under a TFLLR-NH_2_ free condition as a baseline.

We reported that PAR1 activation conducted epithelial-mesenchymal transition (EMT) and lead Snail to move into the nucleus in human gastric cancer [22]. We are now investigating the relationship between the Hippo-YAP pathway and EMT. We prepared knockdown of Snail and YAP in MKN45 and MKN74 cells by transfected each siRNA (Figure S2). In Snail siRNA transfected MKN45/PAR1 and MKN74 cells, we found that Dephosphorylated YAP moved into the nucleus and phpsphorylated Lats1 was inhibited expression by western blot assays, when these cells were treated by TFLLR-NH_2_ (Figure 6A). Subsequently, we analyzed whether EMT was induced by TFLLR-NH_2_, when Hippo-YAP pathway was inhibited. Western blot assays indicated that the EMT markers E-cadherin and fibronectin as well as morphology were unchanged in both MKN45/PAR1 and MKN74 cells treated with C3 and TFLLR-NH_2_ (Figure 6B and 6C). In addition, C3 treatment resulted in cell migration and invasion that was significantly reduced compared to PAR1 activated cells (Figure 6E, 6F and Video S1, S2, S4, S5). Knockdown of YAP as well as the C3 treatment efficiently blocked cell migration, invasion and cell morphology change, and E-cadherin and fibronectin expression levels were not impacted (Figure 6B, 6C, 6E, 6F, and Video S3, S6). Moreover, C3 treatment and knockdown of YAP resulted in Snail not being detected among nuclear proteins (Figure 6B). Furthermore, C3 treatment and knockdown of YAP efficiency blocked increase in side population cells, when MKN45/PAR1 and MKN74 cells were treated by TFLLR-NH_2_ (Figure 6D).

## DISCUSSION

High PAR1 expression was found in tumors including malignant melanoma and breast cancer [27-29] and correlated with invasiveness and motility of numerous cancer cell lines, indicating that PAR1 might act as an oncogene. In addition PAR1 contributes to leukemic stem cell maintenance [30]. In this study, we demonstrate that PAR1 activation induced gastric cancer cells to side population cells which acquired efflux chemotherapy resistance, and PAR1 activity inhibits YAP phosphorylation and increases YAP activity via Rho GTPase. We also showed that PAR1 correlates with Hippo-YAP pathway in mediation of the physiological functions of cancer cell migration and invasion. These data indicated that PAR1 significantly correlated to gastric cancer stem-like cell maintenance and gastric cancer cell invasion through Hippo-YAP signaling pathway in the downstream of PAR1.

CSCs are defined as a group of cells within a tumor that can self-renew and drive tumorigenesis. Cancer cells in a side population are enriched with tumorigenic stem-like cancer cells [10]. We showed that activated PAR1 promoted the propagation of side population cells with efflux drug resistance and had a high tumor-initiating capacity. GPCR signaling correlates with cancer development as both familial and somatic activating mutations of GPCRs have been linked to human cancer [31]. PAR1 is also a member of GPCR. Thrombin's (PAR1 agonist) actions on endothelial cells contribute to vascular development and hemostasis in the mouse embryo [32]. These data and reports suggest that activated PAR1 signaling increases the pool of CSCs.

Several reports have demonstrated that thrombin increases the expression of connective tissue growth factor and Cyr61 which are increased gene expression by the TEAD family [33-35]. Dephosphorylated YAP localizes in the nucleus and function as a transcription coactivators for TEAD family [15]. We showed recently that activated PAR1 triggered the activation of Rho GTPase. And this study shows that activation of PAR1 inhibits Lats1/2 kinase via Rho GTPase, not the ROCK pathway, leading to eventual YAP dephosphorylation, and nuclear localization. We have also shown that when Rho GTPase is inhibited by C3, side population cells which were enriched with CSCs, do not increase as was observed under PAR1 activation do. The Hippo pathway can control its effects on tissue size by the direct regulation of stem cell proliferation and maintenance [36]. We indicate that PAR1 activation suppresses Hippo-YAP pathway via Rho GTPase, and the connection between PAR1 and Hippo signaling pathway increases CSCs proliferation.

**Figure 7:**
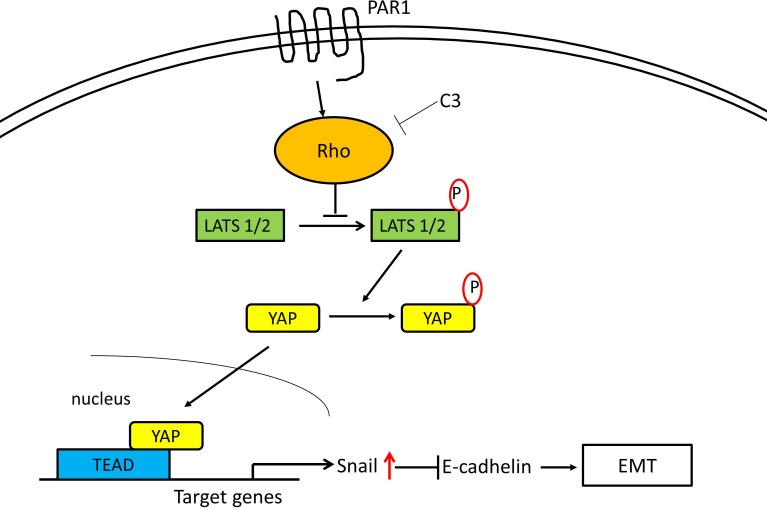
Proposed intracellular transduction mechanisms underlying PAR1-induced inhibition of Hippo-YAP pathway and EMT through Snail Activation of the PAR1 by the selective PAR1-AP TFLLR-NH_2_ results in Rho activation mediating the inhibition of Hippo-YAP cascades leading to EMT via Snail.

We reported that PAR1 activation by thrombin induced epithelial-mesenchymal transition (EMT) via Snail [22]. And YAP and TAZ have also been shown to induce EMT in human breast epithelial cells in a Tead-dependent manner [37]. Lats2 acts as a positive modulator of Snail1 protein level and potentiates its *in vivo* EMT activity [38]. And YAP potentiates Smad signaling to increase expression of the downstream target genes Snail, Slug, and Twist1, which are important transcriptional regulators of EMT [39]. At this time, we have shown that the Hippo-YAP pathway undergoing PAR1 activation is deeply involved with in the induction of EMT. When PAR1 signaling via Rho was inhibited or YAP underwent knockdown, EMT markers E-cadherin and fibronectin expression levels were not changed significantly and cell motility were markedly reduced. Migration is an important initial step in EMT and this decrease in cell motility further underlines the importance of PAR1. When Snail underwent knockdown, we confirmed that YAP was dephosphorylated and localized in nucleus under the influence of TFLLR-NH_2_. These data implicates that the Hippo-YAP signaling pathway caused by activated PAR1 significantly increased with the intranuclear mobility of Snail which was promoted by PAR1 activation, and so this signaling pathway induced EMT in oncogenesis. The pathways established are summarized in the scheme shown in Figure 7.

Elevated YAP nuclear localization is observed in many human cancers [40], but the mechanism upstream (behind) YAP activation in cancer is unknown. The relationship between PAR1 and Hippo pathway clarified by this study may provide a better understanding of the role of YAP activation in gastric cancer. PAR1 is widely expressed in many cell types and has been involved in a wide range of physiological regulation. Degenerated PAR1 signaling is implicated in tumor cell invasion. Activated PAR1 and the Rho signaling pathway can cause unusual cell growth, and ligands of PAR1 are increased under the pathophysiological conditions. For instance, thrombin and PAR1 expression are up-regulated in a tumor microenvironment and invasive cancer cells. We speculate that dephosphorylation of YAP by PAR1 may correlate with cancer invasion and metastasis. We hypothesize that activated PAR1 expression holds the potential for the new predictive factors of chemotherapeutic resistance in the systemic treatment of human gastric cancer, and inhibition of YAP will be a new approach to treat gastric cancer caused by dysregulated PAR1 and its selective agonists.

## MATERIALS AND METHODS

### Reagents

An antibody against PAR1 was purchased from BECKMAN COULTER. Anti-E-cadherin, fibronectin, YAP1, phospho-YAP1 (pYAP1), Lats1 and phospo-Lats1 (pLats1) were purchased from Abcam. Anti-ABCG2, -MRP1, and -P-glycoprotein (P-gp) were purchased from GeneTex. Anti- ribophorin II (RPN2) was purchased from OriGene. Anti-GAPDH was from IMGENEX. The selective PAR1 agonist TFLLR-NH_2_ and PAR1 antagonist SCH79797 was purchased from Tocris Bioscience. We used TFLLR-NH_2_ (EC50: 1.9 μM) at the 20 μM, and SCH79797 (IC50: 70 nM) at the 70 nM. C3 transferase and Y27932 were purchased from Cytoskeleton. We used C3 at the 2 μg/ml and Y27632 at 10 μM. Small interfering RNA (siRNA) directed against PAR1 (5′-AAGGCUACUAUGCCUACUACU-3′) was synthesized by TOYOBO, and siRNA directed against YAP1 (5′-GGUCAGAGAUACUUCUUA-3′) and Snail (5′- CCACAGAAAUGGCCAUGGGAAGGCCUC-3′) were synthesized by Sigma. Control siRNA is a scrambled sequence with no homology in the human genome (Qiagen) is listed as follows: Scrambled, UUCUCCGAACGUGUCACGUdTdT.

### Cell culture and transfection

The human gastric cancer cell lines, MKN45 and MKN74 cells, were obtained from the Riken Cell Bank and we had established PAR1 stable expressing MKN45 cell (MKN45/PAR1) [21]. Cells were cultured at 37°C in 5% CO2 in RPMI-1640 medium (Sigma) containing 10% fetal bovine serum (FBS) (Gibco). Cancer cells seeded before the experiment were gently detached from the surface using Accutase (innovative cell technologies). Endogenous PAR1 is expressed in MKN74, not in MKN45 [21]. MKN45/mock and MKN74 cells served as control cells in this experiment. Ten nM siRNA was introduced into cells by transient transfection with RNAi MAX (Invitrogen) in accordance with the manufacturer's instructions. Four days after the transfection, protein was extracted, and knockdown of target protein was confirmed by western blot assays.

### Western blotting assay

Whole, Cytoplasmic and nuclear cell protein was extracted using RIPA-buffer (Wako) containing 1% protease inhibitor cocktail (Sigma) and NE-PER (Thermo Scientific). Proteins were resolved by SDS-PAGE using a 5-20% SuperSep gel (Wako) and analyzed by Western blot using polyvinylidene difluoride membranes (Millipore) according to the manufacturer's instructions. An enhanced chemiluminescence detection system (Imunostar, Wako) was used to visualize of immunoreactive-bands after the reaction with the HRP-labeled secondary antibody against mouse or rabbit IgG.

### Immunoprecipitation and kinase assay

For the Lats1 kinase assay, both MKN45/PAR1 and MKN74 cells were transfcted with pGEX-KG-GST-YAP (Addgene). Cell lysates were centrifuged for 10 min at 4°C, and the supernatants obtained by centrifugation were incubated with Lats1 anyibodies for 2 h at 4°C, and Dynabeads protein A/G (VERITAS) were added in for 1 h. Immunoprecipitated Lats1 was subjected to a kinase assay in presence of 500 μM cold ATP and GST-YAP as substrate. The phosphorylation of GST-YAP at S127 was determined by immunoblotting using pYAP1 antibody.

### Immunofluorescence

Cultured cells were fixed with 4% paraformaldehyde at room temperature, permeabilized with 0.1% Triton X-100 in PBS and blocked with 3% FBS in PBS. Following overnight incubation at 4°C with primary antibodies, and incubation in the dark with Alexa 633 Fluor dye-labeled secondary antibody, immunofluorescence was detected using a Leica DMLB confocal laser fluorescence microscope (Leica Microsystems).

### Immunohistochemistory

The tumor tissues were routinely fixed in 10% paraformaldehyde and embedded in paraffin. The sections were dewaxed using xylene and rehydrated in graded alcohols. The sections were incubated overnight with the primary antibodies at 4°C in a humidified chamber. The sections were incubated for 60 min at room temperature with labeled-dextran polymer (Envision; Dako). The sections were developed with activated 3′-diaminobenzidinetetrahydrochloride (DAB) for 5 min. The positive cells exhibited the deposition of brown DAB precipitate.

### Cell invasion assay

Invasion of cells through matrigel was determined using a Transwell system (CHEMICON) in accordance with the manufacturer's instructions. Both TFLLR-NH_2_ and C3 were added to the cells (0.5×10^6^ cells/well) in the upper well containing serum-free medium. The A560 nm of the MKN45/mock, MKN74 and MKN45/PAR1 cells cultured under noted conditions were determined and compared using the A560 nm of MKN45/mock and MKN74 cultured under a PAR1 agonist-free condition as a baseline.

### Scratched and time lapse assay

The cells were seeded into 6-well plates to 80-90% confluence and the cell monolayer scratched in a straight line with a 200 μl pipette tip to create a scratch. Then the culture was re-fed with TFLLR-NH_2_. Images were taken at a 0 and 24 h after the scratch to calculate the cell migrationrate. For time-lapse video microscopy, mitotic cells were shaken off the plates and seeded into a six-well plate containing etched coverslips (Bellco) coated with polylysine. Timelapse images were recorded with an Olympus MicroSuite™-B3SV.

### Spheroid colony formation assay

Spheroid colony formation assay were performed in ultra-low attachment 96-well culture dish (Corning). These cells were exposed to TFLLR-NH_2_ for 12 h, and incubated for a minimum of 72 h at 37°C/ 5% CO_2_ and 95% humidity. Then we counted spheroid formation.

### Fluorescence-activated cell sorting of side population

Side population analysis was done as previously described before [41]. Cells were incubated with Hoechst 33342 (Sigma) for 90 min at a final concentration of 5 μg/ml, and analysed by FACS (FACSVantage SE, equipped with FACS DIVA software, version 6.0; BD Biosciences). Dual-wavelength FACS analysis identified a side branch of ‘Hoechst-low’ cells as the side population, further verified by co-adding verapamil (100 μM; Sigma) which results in reduction of the side population size by blocking the dye efflux through multidrug transporter(s).

### Drug resistance assay

Cell viabilities in response to chemotherapeutic induction, and the degrees of cytotoxicity of Cisplatin, 5-FU and Paclitaxel on the cells were measured using cell counting kit CCK-8 (DOJINDO). The number of viable cells is directly proportional to the amount of the formazan dye generated in cells. Animal studies

Pathogen-free, 6-week-old, female BALB/cAJcl-*nu/nu* mice (CLEA Japan) were housed in microisolator cages maintained in a barrier facility. All the mice had a body weight of between 20 and 25 g at the start of treatment, complying with the standards set out in the Guidelines for the Care and Use of Laboratory Animals in the University of Fukui. Each group was composed of five female athymic nude mice. And these nude mice were inoculated intraperitoneally with 1 × 10^6^ cells which were treated under varying conditions. One group was MKN45/mock, MKN45/PAR1 and MKN74 cells as control group. The second and third group were that MKN45/PAR1 and MKN74 cells which were pretreated by TFNLLR-NH_2_ for 24 h together before inoculation. The 4^th^ and 5^th^ group were that MKN45/PAR1 and MKN74 cells which were pretreated by TFNLLR-NH_2_ plus SCH79797 for 24 h together before inoculation. The mice sacrificed 30 days after tumor injection. We examined the size and number tumors in the abdomen. The relative tumor burden in each mouse was calculated by dividing tumor mass weight by total *situs* weight.

### Statistics

Throughout the study, results are presented as mean ± SE. Statistical analysis was performed using SPSS 17.0 (SPSS, Inc.). In the mouse studies, differences between groups were analyzed using the Mann-Whitney rank-sum test. Data were analysed using one-way analysis of variance followed by unpaired Student's *t*-test for caparison between groups. Differences between groups were considered statistically significant at *P* < 0.05. Experiments were performed in duplicate.

## SUPPLEMENTARY FIGURES AND VIDEOS















## References

[1] Visvader JE, Lindeman GJ (2008). Cancer stem cells in solid tumours: accumulating evidence and unresolved questions. Nat Rev Cancer.

[2] Goodell MA, Brose K, Paradis G, Conner AS, Mulligan RC (1996). Isolation and functional properties of murine hematopoietic stem cells that are replicating *in vivo*. J Exp Med.

[3] Britton KM, Eyre R, Harvey IJ, Stemke-Hale K, Browell D, Lennard TW, Meeson AP (2012). Breast cancer, side population cells and ABCG2 expression. Cancer Lett.

[4] Haraguchi N, Inoue H, Tanaka F, Mimori K, Utsunomiya T, Sasaki A, Mori M (2006). Cancer stem cells in human gastrointestinal cancers. Hum Cell.

[5] Salcido CD, Larochelle A, Taylor BJ, Dunbar CE, Varticovski L (2010). Molecular characterization of side population cells with cancer stem cell-like characteristics in small-cell lung cancer. Br J Cancer.

[6] Wang J, Guo LP, Chen LZ, Zeng YX, Lu SH (2007). Identification of cancer stem-like side population cells in human nasopharyngeal carcinoma cell line. Cancer Res.

[7] Chiba T, Kita K, Zheng YW, Yokosuka O, Saisho H, Iwama A, Nakauchi H, Taniguchi H (2006). Side population purified from hepatocellular carcinoma cells harbors cancer stem-like properties. Hepatology.

[8] Ning ZF, Huang YJ, Lin TX, Zhou YX, Jiang C, Xu KW, Huang H, Yin XB, Huang J (2009). Subpopulations of stem-like cells in side population cells from the human bladder transitional cell cancer cell line T24. J Int Med Res.

[9] Reya T, Morrison SJ, Clarke MF, Weissman IL (2001). Stem cells, cancer, and cancer stem cells. Nature.

[10] Patrawala L, Calhoun T, Schneider-Broussard R, Zhou J, Claypool K, Tang DG (2005). Side population is enriched in tumorigenic, stem-like cancer cells, whereas ABCG2+ and ABCG2- cancer cells are similarly tumorigenic. Cancer Res.

[11] Kabashima A, Higuchi H, Takaishi H, Matsuzaki Y, Suzuki S, Izumiya M, Izuka H, Sakai G, Hozawa S, Azuma T, Hibi T (2009). Side population of pancreatic cancer cells predominats in TGF-beta-mediated epithelial to mesenchymal transition and invasion. Int J Cancer.

[12] Pan D (2007). Hippo signaling in organ size control. Genes Dev.

[13] Zhao B, Li L, Lei Q, Guan KL (2010). The Hippo-YAP pathway in organ size control and tumorigenesis: an updated version. Genes Dev.

[14] Lei QY, Zhang H, Zhao B, Zha ZY, Bai F, Pei XH, Zhao S, Xiong Y, Guan KL (2008). TAZ promotes cell proliferation and epithelial-mesenchymal transition inhibited by the hippo pathway. Mol Cell Biol.

[15] Zhao B, Ye X, Yu J, Li L, Li W, Li S, Yu J, Lin JD, Wang CY, Chinnaiyan AM, Lai ZC, Guan KL (2008). TEAD mediates YAP-dependent gene induction and growth control. Genes Dev.

[16] Zhao B, Li L, Tumaneng K, Wang CY, Guan KL (2010). A coordinated phosphorylation by Lats and CK1 regulates YAP stability through SCF(β-TRCP). Genes Dev.

[17] Camargo FD, Gokhale S, Johnnidis JB, Fu D, Bell GW, Jaenisch R, Brummelkamp TR (2007). YAP1 increases organ size and expands undifferentiated progenitor cells. Curr Biol.

[18] Dong J, Feldmann G, Huang J, Wu S, Zhang N, Comerford SA, Gayyed MF, Anders RA, Maitra A, Pan D (2007). Elucidation of a universal size-control mechanism in Drosophila and mammals. Cell.

[19] Zhao B, Wei X, Li W, Udan RS, Yang Q, Kim J, Xie J, Ikenoue T, Yu J, Li L, Zheng P, Ye K, Chinnaiyan A, Halder G, Lai ZC, Guan KL (2007). Inactivation of YAP oncoprotein by the Hippo pathway is involved in cell contact inhibition and tissue growth control. Genes Dev.

[20] Pan D (2010). The Hippo signaling pathway in development and cancer. Dev Cell.

[21] Fujimoto D, Hirono Y, Goi T, Katayama K, Matsukawa S, Yamaguchi A (2010). The activation of Preteinase-Activated Receptor-1 (PAR1) mediates gastric cancer cell proliferation and invasion. BMC Cancer.

[22] Otsuki T, Fujimoto D, Hirono Y, Goi T, Yamaguchi A (2014). Thrombin conducts epithelial-mesenchymal transition via protease-activated receptor-1 in human gastric cancer. Int J Oncol.

[23] Honma K, Iwao-Koizumi K, Takeshita F, Yamamoto Y, Yoshida T, Nishino K, Nagahara S, Kato K, Ochiya T (2008). RPN2 gene confers docetaxel resistance in breast cancer. Nat Med.

[24] Fujimoto D, Hirono Y, Goi T, Katayama K, Matsukawa S, Yamaguchi A (2013). The activation of proteinase-activated receptor-1 (PAR1) promotes gastric cancer cell alteration of cellular morphology related to cell motility and invasion. Int J Oncol.

[25] Dupont S, Morsut L, Aragona M, Enzo E, Giulitti S, Cordenonsi M, Zanconato F, Digabel JL, Forcato M, Bicciato S, Elvassore N, Piccolo S (2011). Role of YAP/TAZ in mechanotransduction. Nature.

[26] Chan EH, Nousiainen M, Chalamalasetty RB, Schafer A, Nigg EA, Sillje HH (2005). The Ste20-like kinase Mst2 activates the human large tumor suppressor kinase Lats1. Oncogene.

[27] Massi D, Naldini A, Ardinghi C, Carraro F, Franchi A, Paglierani M, Tarantini F, Ketabchi S, Cirino G, Hollenberg MD, Geppetti P, Santucci M (2005). Expression of protease-activated receptors 1 and 2 in melanocytic nevi and malignat melanoma. Hum Pathol.

[28] Even-Ram S, Uziely B, Cohen P, Grisaru-Granovsky S, Maoz M, Ginzburg Y, Reich R, Vlodavsky I, Bar-Shavit R (1998). Thrombin receptor overexpression in malignant and physiological invasion processes. Nat Med.

[29] Hernandez NA, Correa E, Avila EP, Vela TA, Perez VM (2009). PAR1 is selectively over expressed in high grade breast cancer patients: a cohort study. J Transl Med.

[30] Baumer N, Krause A, Kohler G, Lettermann S, Evers G, Hascher A, Baumer S, Berdel WE, Muller-Tidow C, Tickenbrock L (2014). Proteinase-Activated Receptor 1 (PAR1) regulates leukemic stemm cell functions. PLoS One.

[31] Dorsam RT, Gutkind JS (2007). G-protein-coupled receptors and cancer. Nat Rev Cancer.

[32] Griffin CT, Srinivasan Y, Zheng YW, Huang W, Coughlin SR (2001). A role for thrombin receptor signaling in endothelial cells during embryonic development. Science.

[33] Chambers RC, Leoni P, Blanc-Brude OP, Wembridge DE, Laurent GJ (2000). Thrombin is a potent inducer of connective tissue growth factor production via proteolytic activation of protease-activated receptor-1. J Biol Chem.

[34] Walsh CT, Radeff-Huang J, Matteo R, Hsiao A, Subremaniam S, Stupack D, Brown JH (2008). Thrombin receptor and RhoA mediate cell proliferation through integrins and cysteine-rich protein 61. FASEB J.

[35] Lai D, Ho KC, Hao Y, Yang X (2011). Taxol resistance in breast cancer cells is mediated by the hippo pathway component TAZ and its downstream transcriptional targets Cyr61 and CTGF. Cancer Res.

[36] Zhao B, Tumaneng K, Guan KL (2011). The Hippo pathway in organ size control, tissue regeneration and stem cell self-renewal. Nat Cell Biol.

[37] Diepenbruck M, Waldmeier L, Ivanek R, Berninger P, Arnold P, van Nimwegen E, Christofori G (2014). Tead2 expression levels control the subcellular distribution of Yap and Taz, zyxin expression and epithelial-mesenchymal transition. J Cell Sci.

[38] Zhang K, Rodriguez-Aznar E, Yabuta N, Owen RJ, Mingot JM, Nojima H, Nieto MA, Longmore GD (2012). Lats2 kinase potentiates Snail1 activity by promoting nuclear retention upon phosphorylation. EMBO J.

[39] Zhang H, von Gise A, Liu Q, Hu T, Tian X, He L, Pu W, Huang X, He L, Cai CL, Camargo FD, Pu WT, Zhou B (2014). Yap1 is required for endothelial to mesenchymal transition of the atrioventricular cushion. J Biol Chem.

[40] Chan SW, Lim CJ, Guo K, Ng CP, Lee I, Hunziker W, Zeng Q, Hong W (2008). A role for TAZ in migration, invasion, and tumorigenesis of breast cancer cells. Cancer Res.

[41] Chen J, Hersmus N, Van Duppen V, Caesens P, Denef C, Vankelecom H (2005). The adult pituitary contains a cell population displaying stem/progenitor cell and early embryonic characteristics. Endocrinology.

