# Supplementary material for: PAR1 participates in the ability of multidrug resistance and tumorigenesis by controlling Hippo-YAP pathway
Source: Oncotarget. 2015 Sep 28;6(33):34788–99. doi: 10.18632/oncotarget.5858 (PMC4741490; doi:10.18632/oncotarget.5858)
Supplement: Supplementary file 1 [file oncotarget-06-34788-s001.pdf]

PAR1 participates in the ability of multidrug resistance and tumorigenesis by controlling Hippo-YAP pathway

Supplementary Material

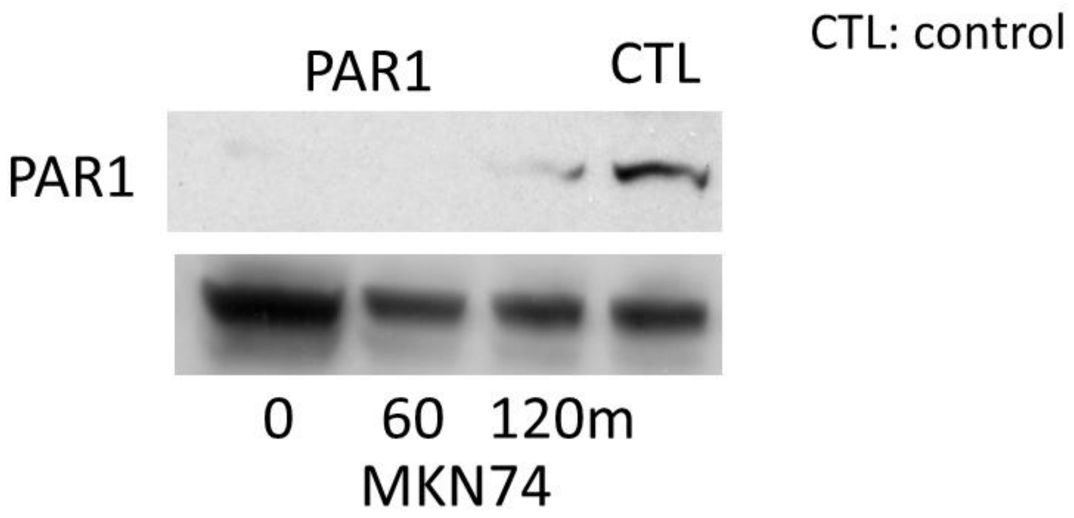

Figure S1.

Evidence for knockdown of a target protein caused by transfection with siRNA specific to the gene. MKN74 cell were transfected with scrambled siRNA (control); GAPDH was used as loading control. PAR1 siRNA followed by western blot assays using anti-PAR1 antibodies.

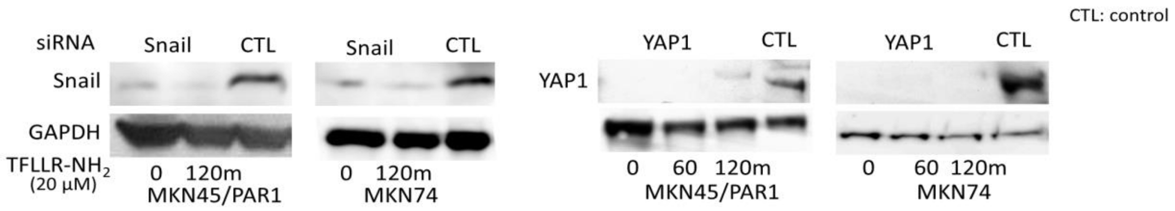

Figure S2

Evidence for knockdown of a target protein caused by transfection with siRNA specific to the gene. MKN45/PAR1 and MKN74 cells were transfected with scrambled siRNA (control); GAPDH was used as loading control. Snail, and YAP1 siRNA followed by western blot assays using anti-Snail and anti-YAP1 antibodies.

Video S1. This file contain example of the movie, which can be viewed with Windows Media Player, were generated during analysis of MKN45/PAR1 cells migration under treating the TFLLR-NH_2. The red arrow indicates the point where cells have often migrated.

Video S2. This file contain example of the movie, which can be viewed with Windows Media Player, were generated during analysis of MKN45/PAR1 cells migration pretreated with C3 for 5 h and then incubated with TFLLR-NH_2.

Video S3. This file contain example of the movie, which can be viewed with Windows Media Player, were generated during analysis of MKN45/PAR1 cells migration, when these cell's YAP1 was knocked down by siRNA and then incubated with TFLLR-NH_2.

Video S4. This file contain example of the movie, which can be viewed with Windows Media Player, were generated during analysis of MKN74 cells migration under treating the TFLLR-NH_2. The red arrow indicates the point where cells have often migrated.

Video S5. This file contain example of the movie, which can be viewed with Windows Media Player, were generated during analysis of MKN74 cells migration pretreated with C3 for 5 h and then incubated with TFLLR-NH_2.

Video S6. This file contain example of the movie, which can be viewed with Windows Media Player, were generated during analysis of MKN74 cells migration, when these cell's YAP1 was knocked down by siRNA and then incubated with TFLLR-NH_2.
